# The Association of Dietary Intake of Calcium and Vitamin D to Colorectal Cancer Risk among Iranian Population

**DOI:** 10.31557/APJCP.2019.20.9.2825

**Published:** 2019

**Authors:** Payam Hosseinzadeh, Mohammad Javanbakht, Mahdi Alemrajabi, Ali Gholami, Bahareh Amirkalali, Masoudreza Sohrabi, Farhad Zamani

**Affiliations:** 1 *Gastrointestinal and Liver Diseases Research Center (GILDRC),*; 3 *Firoozgar Hospital, FCRDC, Iran University of Medical Sciences (IUMS),*; 2 *Nephrology and Urology Research Center, Baqiyatallah University of Medical Sciences, Tehran,*; 4 *Noncommunicable Diseases Research Center,*; 5 *Department of Epidemiology and Biostatistics, School of Public Health, Neyshabur University of Medical Sciences, Neyshabur, Iran.*

**Keywords:** Cancer, Vitamin D, calcium, rectal

## Abstract

**Background:**

Vitamin D and Calcium have a possible protective impact versus rectal neoplasm. Vitamin D, an important nutrient, is vital to regulate the absorption of calcium and bone mineralization; nevertheless, in a case-control study in Iran, we investigated the relationship among the dietary intake of vitamin D and calcium with the hazard of rectal neoplasm.

**Methods:**

363 subjects (162 cases and 201 controls) participated in the case- control Study from March 2017 to November 2018. Dietary intake of Calcium and Vitamin D was calculated using a 148-items food-frequency questionnaire.

**Results:**

Since altering the strong confounding agents, the multivariate risk proportion within the dietary vitamin D intake was OR=0.2, 95%CI 0.1-0.5, P-value <0.001 among cases. There was no association in case of calcium and rectal cancer.

**Conclusions:**

Taken together, a possible reduction in the hazard of rectal neoplasm with dietary intake of Vitamin D within Iranian patients was observed.

## Introduction

Colorectal cancer (CRC) is a typical neoplasm, which widely recognized as a main agent of mortality in the worldwide (Ferlay et al., 2013). Some portion of the expansion in rectal cancer rate might be clarified by latest modification in lifestyle variables, counting dietary templates in Iranian people (Azadeh et al., 2008)

In spite of thorough research to distinguish explicit dietary agents for CRC risk, just a couple of dietary agents, for example, fiber, folic acid and omega-3 have been indicated or may be reliably to be related with CRC hazard (Besharati et al., 2018). Most of the past investigations, but, were led in Western governments (Key et al., 2012; Shin et al., 2006). The particular dietary habits of Iranian individuals as a part of middle-east populations could help us understand the role of diet in the progress of CRC (Besharati et al., 2017). Due to changing ecological conditions, both calcium and vitamin D ,which are micronutrients, may have an insufficient intake in Iran; A scope of evidence like low consumption of dairy products and less exposure to sunlight due to the risk of skin cancer have implicated Calcium and vitamin D insufficiency cause the increased liability to various gastrointestinal disorders, including RC (Ferguson, 2018) 

Vitamin D, a basic supplement that is significant for bone mineralization and regulates the absorption of calcium (Brown and Slatopolsky, 1999), Also has impacts beyond bone and calcium homeostasis. New evidence recommends a conceivable interactive efficacy of calcium and vitamin D on the hazard of recurrent adenoma (Ma et al., 2011). Several mechanisms might clarify the possibly defensive impacts of calcium and vitamin D versus CRC, involving the joint of secondary bile acids and fatty acids that practice as a motive for colon and intestinal lumen epithelial cells (Harris et al., 2004; Feldman et al., 2014).

Furthermore, recent studies propose a lot more extensive scope of biological functions of vitamin D, which include the regulation of proliferation, disparity, apoptosis, inhibition of angiogenesis, and immunity in prevention of colorectal cancer (Deeb et al., 2007; Feldman et al., 2014). Most of the observational examinations show that the increased calcium and vitamin D intake lead to a diminished hazard of CRC (Flood et al., 2005; McCullough et al., 2003). One study suggested that a superior status of vitamin D may ameliorate during recognition and therapy survival from rectal cancer (Giovannucci, 2007). Exploratory and epidemiological investigations indicate that vitamin D has a defensive role in colorectal carcinogenesis; however, evidence is not certain (McCullough et al., 2019). To support more, reversed connection among these measures and the hazard of CRC or adenoma have been demonstrated through observational studies of vitamin D intake and serum levels of 25-hydroxy vitamin D (Avenell et al., 2012). 

High intake of vitamin D represses experimental carcinogenesis (Feldman et al., 2014). Even in animals that are vitamin D- replete trials of vitamin D supplementation, a reduction in the prevalence of CRC in relationship with supplementation has not been demonstrated (Wactawski-Wende et al., 2006; Avenell et al., 2012). However, some restrictions such as little quantities of events (Lappe et al., 2007; Avenell et al., 2012), low vitamin D doses (Wactawski-Wende et al., 2006), and relatively short follow-up courses for invasive neoplasm ends were applied (Lappe et al., 2007; Wactawski-Wende et al., 2006).

High intake of calcium is likewise connected with lesser hazards of CRC. It seems that the increase of calcium in dietary inhibit the large-bowel carcinogenesis in animal models, also the epidemiologic studies have demonstrated the higher calcium intake causes the lesser hazards of CRC and adenomas (Huncharek et al., 2009). Preliminaries of calcium supplementation for adenoma avoidance have shown diminished hazards (Chu et al., 2011). In spite of the fact that outcomes are relatively blended, a report of decrease about 10 -1 % in the occurrence of CRC was presented through the analysis of 10 associate studies ,which evaluated the dietary consumption and total calcium intake (Cho et al., 2004), The proposal that increased intake of calcium ,which help the prevention of CRC, led to randomized clinical trial and showed the lowered frequency of recurrent colorectal polyps to some degree after taking the calcium supplementation (Bonithon-Kopp et al., 2000) and a other study exhibited that this protection was restricted to subjects with greater endogenous vitamin D levels (Grau et al.,2003). Conversely, in a randomized, multi-center, placebo-controlled trial, involving 2,259 participants the impacts of daily supplementation with calcium (1,200 mg), vitamin D (1,000 IU), or both after removing the colorectal adenomas were evaluated. Any of the cures did not essentially increase the hazard of recurrent colorectal adenomas more than 3 to 5 years (Baron et al., 2015).

Therefore, in this case control study, we performed a connection of the calcium and vitamin D intake to CRC hazard among Iranian population.

## Materials and Methods

*Study Design*

Current research is a case-control study consists of 162 cases aged 40–80, which were matched with 201 controls and recruited between march 2017 and November 2018. Frequent matching was performed in this study; in the following, for cases included in the study, controls from the same sex and age groups (40-49, 50-59, 60-69 and ≥ 70 years old) were selected. Cases were patients diagnosed with non-metastatic rectum cancer who were in chemo-radiation waiting list of Cancer Institute of Emam-Khomeini and Firoozgar Hospitals in Tehran, Iran. These patients were detected in a parallel phase III clinical trial (Clinical trial number: IRCT2016061118745N8) that were conducted by our research team in the mentioned hospitals. The accident instances were identified from the announcements of the initial detection of CRC (International Classification of Diseases 10th revision rubric C19, C20, C21.8) (Bassett et al., 2013). Controls were out patients with any cancer or serious diseases, referring to the above-mentioned hospitals. We investigated the relationship of some independent variables to the chance of getting cancer (Table 1). 

Exclusion criteria for cases were Patients with the age of less than 40 and more than 80 years old. Patients with metastatic cancer of the rectum or history of colorectal cancer in their family, Liver problems (serum aspartate aminotransferase (AST) or alanine aminotransferase (ALT) concentrations greater than100 IU/L), Serious acute or chronic heart disease, Metastatic brain or lung, Bowel obstruction, Kidney problems (Glomerular Filtration Rate (GFR) ≤ 30 or serum creatinine concentrations greater than 1.7 mg/dl), Aids/Hepatitis, Abnormal blood cell count introduced by white blood cell (WBC) counts greater than10,000 cells/L, hemoglobin levels less than 10 mg/dl or platelet counts less than 15,000/mcl or greater than 400,000/mcl, Specific drug regimen or consuming supplements that contained calcium or/and vitamin D, Smoking and/or alcoholic Drinking were excluded from the study. All participants signed the consent form. Both of the study protocol and consent form were approved by The Iran University of Medical Sciences (IUMS) Ethics Committee (No.26713).

**Table 1:** Characteristics of Study Population (n=363)

Study Group	Case (n=162)		Control (n=201)	
Variable	n	%	n	%
Age				
40-49 years old	68	42	87	43.2
50-59 years old	43	26.5	58	28.9
60-69 years old	41	25.3	45	22.4
70 years old	10	6.2	11	5.5
Sex				
Female	68	42	93	46.3
Male	94	58	108	53.7
Job				
Active	114	71	167	83
Passive	48	29	34	17
Marital Status				
Single/Widow/Divorced	45	27	53	26
Married	117	73	148	74
Education				
< 12 years	89	57	137	70
≥12 years	73	43	64	30
Family Size				
≤ 3 persons	29	19	55	27
˃ 3 persons	33	81	146	73
Income				
< 500$ per month	65	41	101	51
$ per month	97	59	100	49
Physical Activity				
Light	134	82	96	48
Moderate and more	28	18	105	52
BMI*				
<25	56	34	56	28
≥25	106	66	145	72

*, Body Mass Index

**Table 2:** Odds Ratio (OR) Estimates of Rectal Cancer Based on the Univariate Logistic Regression

Study Group	Case (n=162)	Control (n=201)	OR (%95 CI)	P-value
Variable				
Job				
Active	114	167	2.1 (1.3-3.4)	0.004
Passive	48	34		
Marital Status				
Single/Widow/ Divorced	45	53	0.9 (0.6-1.5)	0.764
Married	117	148		
Educational years				
< 12 years	89	137	1.8 (1.1-2.7)	0.01
≥12 years	73	64		
Family Size				
≤ 3 persons	29	55	1.7 (1.1-2.9)	0.034
˃ 3 persons	33	146		
Household Income				
< 500$	65	101	1.5 (0.9-2.3)	0.054
≥500$	97	100		
Physical Activity				
Light	134	96	0.2 (0.1-0.3)	<0.001
Moderate and more	28	105		
BMI*				
<25	56	56	0.7 (0.5-1.1)	0.169
≥25	106	145		
Calcium				
Low intake†	110	122	1.3 (0.8-2.1)	0.155
Adequate intake‡	52	79		
Vitamin D				
Low intake†	58	39	0.4 (0.2-0.6)	<0.001
Adequate intake‡	104	162		

*, Body Mass Index; †, Daily intake of Calcium/Vitamin D less than Dietary Reference Intakes (DRIs), Data from Krause’s Food & Nutrition Therapy (Edition14); ‡, Adequate daily intake of Calcium/Vitamin D based on Dietary Reference Intakes (DRIs), Data from Krause’s Food & Nutrition Therapy (Edition14); P-value≤0.05 is statistically significant.

**Table 3:** Adjusted Analysis of Calcium/Vitamin D Family Based on Backward Multiple Linear Regression model

Variable	Beta	OR (CI)	*P-value*
Calcium	0.34	1.4 (0.8-2.2)	0.17
Vitamin D*	-1.25	0.2 (0.1-0.5)	<0.001

*P-value≤0.05 is statistically significant

*Data Collection and Nutrient Analysis*

Data was gathered using a set of instruments, including a socio-demographic questionnaire (i.e. age, gender, marital status, educational years, and household income). In addition, a semi quantitative food frequency questionnaire (FFQ) was administered. The validity of the Iranian version of a standard FFQ was assessed by Kelishadi et al., (2003), Kelishadi et al., (2004) and Esfahani et al., (2010).

The FFQ asked contributors to evoke last year’s high alternation of consumption. The FFQ also weighed the mean quantity consumed every day. Samples and pictures of various sizes of the FFQ section are provided to assist contributors in estimating the mean quantity of food consumed every day. The mean alteration of food intake every week and month of FFQ was transformed to a daily intake. Once the mean daily intake of every food kind was calculated, the nutrient intake every day was computed by the following formula: nutrient intake per day = ∑ (daily intake of food (consumption frequency) × amount of nutrient in the food × amount of consumed food). The nutritional composition of foods consumed (calcium, vitamin D) was showed from the Iranian food composition table (Mostafavi et al., 2017). Nutrient composition data for calcium and vitamin D were derived from the NUT4 database as utilized for the same prior papers (Hosseinzadeh et al., 2013a; Hosseinzadeh et al., 2013b). 

*Statistical analysis*

Statistical analyses were applied utilizing SPSS for Windows (Version 22. Chicago, SPSS Inc.). Descriptive statistics included frequencies, percentages, ranges, means, and standard deviations (SD). The univariate examination was used to investigate the relationship between rectal cancer and patients’ attributes including age, sex, income, education level, family size, work position, marital status, physical activity, Body Mass Index (BMI) and calcium/vitamin D intake. After adjusting the impact of other elements, we assessed the relationship between calcium/vitamin D intake and rectum cancer by numerous strategic relapse models with the retrogressive strategy. The significance level was set at P < 0.2 in univariate analyses and P < 0.05 for multiple analyses. The odds ratios (ORs) and 95% confidence intervals (CI) reported for evaluated variables. 

## Results

Out of the 200 cases who agreed to participate in this study and provided informed consent, 6 cases were removed because of far-fetched energy intakes (<500 kcal/ day or >4,000 kcal/day), 13 cases were excluded because they provided incomplete FFQ. Contributors who has presented a detect of heart diseases, diabetes, all types of cancers and other serious diseases, consumption of calcium/vitamin D supplements or regular non-steroidal anti-inflammatory drugs (NSAID) (n = 11) were excluded. Also, as none of the cases must have alcohol drinking or smoking history, 8 participants who were reported with these criteria, excluded from our study. Therefore, 162 patients with rectal cancer were included in the analyses. The patients’ demographic and life style characteristics are displayed in Table 1.The mean age of cases was 46.2 ± 12.6 (range: 41–76) years old and controls’ was 43.4 ± 13.6 (range: 40–72) years old. The majority of cases were men (58%) and overweight or obese (%66). Based on the univariate analysis, no significant differences were observed in marital status, BMI, and calcium between controls and cases with rectal cancer (P_value>0.2, Table2). We observed job status, educational years, family size, household income, physical activity, and vitamin D have a significant difference between cases and controls (P-value≤ 0.2, Table 2). 

We utilized various calculated relapse models with backward method to evaluate the independent effect of the Calcium/vitamin D on the rectal cancer. According to this model after adjusting for other factors, rectal cancer was just fundamentally associated with vitamin D intake (OR=0.2, 95%CI 0.1-0.5, P_value<0.001). The association between rectal cancer and calcium was not statistically significant (P_ value<0.1, Table 3).

## Discussion

In the present study, the dietary intake of Vitamin D and Calcium was considered by assessing the intake of 148 food items related to rectal cancer in a FFQ. In our observational study, a protective relationship between intake of dietary vitamin D and the risk of rectal cancer in the association of lifestyle factors with cancer have been observed.

Several investigations were done on different animal models of CRC and a protective role of vitamin D is supported in them. In mouse models of CRC induced by exogenous carcinogen, the use of calcitriol or vitamin D obstrcuts the neoplastic activity (Hummel et al., 2013; van Harten-Gerritsen et al., 2015). In a human CRC (MC26) xenograft model, mice fed sufficient vitamin D have smaller tumors than those fed deficient vitamin D (Tangpricha et al., 2005). Finally, a mouse model of vitamin D receptor knockout (VDR), contrasted to wild-type and heterozygous mice, has indicated elevated markers of cell proliferation and oxidative stress in offspring and colon and supports the anti-neoplastic impact of VDR in large intestine (Kallay et al., 2001)

In a randomized, double-blind, placebo-controlled trial postmenopausal women got (1,000 mg of elemental calcium and 400 IU of vitamin D_3_), the impact on the frequency of colorectal cancer was not seen. Along these lines, the protective role of vitamin D intake was not supported by interventional component of this study. Furthermore, the epidemiological data on duration, although restricted, showed that it might take at least 10 years to emerge the effect of calcium and vitamin D intakes (Prentice et al., 2005). Thus, this randomized trial was likely lacking in testing the vitamin D colorectal cancer hypothesis.

In a few examinations an opposite connection among vitamin D intake and hazard of CRC has been observed (Grant and Garland, 2004). Also, two different researchers have assessed the relationship of calcium and vitamin D supplement intake with neoplasm outbreak. In patients undergoing vitamin D, the Nebraska trial detected the lesser outbreak of neoplasm as well as calcium than with placebo (P < 0.03), though the record trial found no correlation. However, neither of the studies was intended to distinguish the relationship of supplement utilize with CRC prevalence as the early endpoint (Giovannucci, 2007)

The results reported from dietary calcium intake showed no association between calcium and RC risk in our study. However, some studies have found that Calcium has some neoplasm inhibitory impacts, containing binding to cancer-causing bile acid and ionized fatty acid. Calcium may likewise advance the differentiation and controlling development of cells of colon via attaching to calcium identifying receptors (Lamprecht and Lipkin, 2003). There is an inconsistency among calcium and CRC hazard based on Epidemiological evidence; a review of case–control and cohort studies along with two other trials (Hartman et al., 2005; Bonithon-Kopp et al., 2000) did not revealed a protective effect on CRC hazard. Conversely, a French cohort study including 10 prospective investigation indicated a significant protective effect of calcium on colorectal cancer risk. The outcomes revealed a notable reduced hazard of CRC in the individuals who took dietary calcium more than 700 mg/day in comparison with those who took less than 500 mg/day. In present research, the median of dietary calcium intake was 420 mg/day, which is lower than the medians in the former researches (674–1,051 mg/day). Moreover, supplementation with calcium was stated in some studies, which may increase the effect of calcium intake in patients (Cho et al., 2004)

Among various imminent investigations, many found a converse relationship with total intake of calcium, ie, dietary intake along with supplementation with calcium (McCullough et al., 2003; Shin et al., 2006). Nevertheless, the relationship with dietary intake, such as consumption of food and beverages, were not significant in many investigations, basically as a result of the limited amount of dietary calcium intake (Cho et al., 2004).

Therefore, the supportive impact of calcium is mostly among people whom intake the high amount of calcium in their food regiments (Wallace et al., 2004). The other point in the correlation among the hazard of CRC and food calcium was if the supportive impact of calcium is related to calcium or dairy products. Milk, as a diary product, consists of high amount of dietary calcium in USA (Subar et al., 1998), while Cheese and vegetables are considered as the essential origin of calcium in Iran (Omidvar et al., 2015) which have less valuable calcium content than milk. Actually more than 60 percent of people in the Eastern Hemisphere like middle-east residents suffering from lactase deficiency, the enzyme of absorption and digestion of lactose, and consequently have lactose intolerance disorders, a problem that is a disorder and is more or less common in some tribes. This disorder is a noteworthy supporter of numerous individuals refusing to eat milk on this part of the planet, which includes our country. Accordingly, distinguishing the impact of calcium by various dietary sources in different population might be complex.

The diet of the participants in the present research was characterized by taking low amount of dietary calcium and relatively high amount of non-dietary vitamin D from sun exposure, which represents the middle-east diet.

Mean dietary calcium and vitamin D intake in Iran is rather low in comparison to that in Western countries. This finding may show that even this intake may be still too low to decrease the general risk of rectal cancer in this population. Nevertheless, generation of endogenous vitamin D in the cutaneous by sun disposal may have been confused by this finding. In this regard, an ecologic study in Japan discovered an opposite relationship among colorectal cancer mortality and prefectural amount of solar exposure (Mizoue, 2004). As a result, the data at the individual level is required (Otani et al., 2007). One benefit of our population is that, due to the food origin were various, food calcium from cheese and vegetables and dietary vitamin D and risk association with sun exposure could be separately explored. 

*Powers and Limitations*

The strength of our study includes the collection of data on diet and potential confounding variables, including smoking, BMI, physical activity, and other dietary factors. As a result, we were able to finally adjust for prospective confounding factors for RC through data on diet and lifestyle variables. Also, the validity of these dietary patterns has earlier been revealed to be reasonable (Theodoratou et al., 2008).

However, we cannot rule out all confounding factors, including recall and selection bias. Given the potential for aforementioned bias in case-control studies and the limited amount of these studies in middle-east, there is a definite need for further research into incident adenoma and calcium and/or vitamin D intake. 

There are several constraints to this research. First, the FFQ contained 148 food products, which placed a significant strain on the participants. Second, dietary evaluation is also complicated and FFQs are not a perfect measure of regular dietary intakes. Consequently, intakes of certain ingredients including Dairy products, fruit and vegetables may have been underestimated due of difficulty in the accurately recall of usual intakes. The misreporting and misclassification may still have decreased the capacity to detect associations and resulting in residual confounding. Residual confounding from the use of the FFQ is likely to have influenced both the assessment of nutrients intake and the adjustments taken for other prospective dietary confounders.

Although replication by additional research is required, these results indicate interplay of the immune system

and vitamin D status in obstructing the tumorigenesis of rectal cancer. Furthermore, in rectal cancer patient survival analyzes, a possible interaction between tumor immunity status and vitamin D status may require further inquiry. Based on the inflammatory processes and complicated immune, which are involved in development of rectal cancer and controlled by vitamin D, it was introduced that future epidemiological researches, if possible, measure inflammatory markers and vitamin D for multiple times and also, use of mediation analysis to conduct study the function of inflammation as a mediator among rectal cancer and vitamin D.

In conclusion, we discovered a reduced rectal cancer risk with appropriate dietary vitamin D intake among patients with rectal neoplasm, although there was no association with dietary calcium intake. Contrarily, the impact of vitamin D intake on rectal neoplasm avoidance is debatable, mainly owing to three factors: the gradual growth of rectal cancer, the confounding impacts induced by exposure to sunlight, outdoor exercises, body mass index, intake of calcium and dairy products, etc. Lastly, surveys of genome-wide gene-environment and next generation sequencing interactions are likely to clarify the functions of relationship among vitamin D and rectal neoplasm. In addition, more study is required to ascertain the optimal levels and intakes of this vitamin to lower the risk of rectal cancer.
